# P-2080. Impact of the Implementation of Multiplex Gastrointestinal PCR Panel on Antimicrobial Use for Infectious Diarrhea

**DOI:** 10.1093/ofid/ofae631.2236

**Published:** 2025-01-29

**Authors:** Blake R Mangum, Richard D Smith, Matthew Sandruck, J Kristie Johnson, Paul Luethy, Kimberly C Claeys

**Affiliations:** University of Maryland School of Pharmacy, Baltimore, Maryland; University of Maryland Medical Center, Baltimore, Maryland; University of Maryland School of Pharmacy, Baltimore, Maryland; University of Maryland School of Medicine, Baltimore, Maryland; University of Maryland School of Medicine, Baltimore, Maryland; University of Maryland Baltimore, Baltimore, Maryland

## Abstract

**Background:**

Rapid molecular methods, such as BioFire FilmArray™ Gastrointestinal (GI) Panel, have become common in clinical practice. The GI Panel identifies 22 GI pathogens in approximately 1 hour. We evaluated antimicrobial prescribing before and after the implementation of the GI Panel to see the impact on appropriate prescribing for infectious diarrhea and to identify quality improvement areas for antimicrobial stewardship moving forward.

**Figure 2:**
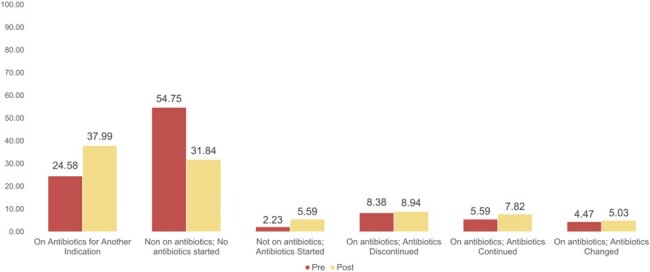
Percent Type of Antibiotic Change After Result of Diagnostic Testing

**Methods:**

This retrospective cohort analysis compared antimicrobial prescribing practices for infectious diarrhea one year before and after the implementation of the BioFire GI Panel at the University of Maryland Medical Center (UMMC) in August 2021. Prior to GI Panel implementation at UMMC, the major diagnostic tests for infectious diarrhea were stool culture, ova and parasite smear, and a parasite PCR. Cases were randomly selected with an even distribution from each month from August 2019 to August 2022. Appropriateness of empiric and definitive management was assessed based on diarrhea type (bloody, acute, or prolonged), the patient's immune status, travel history, clinical presentation, and antibiotic selection.

**Results:**

353 patients were included: 179 pre-GI panel implementation and 174 post-implementation. Empiric antibiotic therapy for infectious diarrhea was started in 47/179 (26.3%) of the pre-panel group and 50/174 (28.7%) of the post-panel (*P* = 0.601). The diagnostic tests resulted in positive organism identification in 10% of patients in the pre-group, while positive identification increased to 19.4% post-group (P = 0.012). When an organism was identified, 12/33 (66.7%) of the post-panel group received antibiotic therapy, while only 6/18 (33.3%) of the pre-panel group received antibiotics (P = 0.83)

**Conclusion:**

Our analysis showed that many patients did not have antimicrobial therapy for infectious diarrhea, which was impacted by the diagnostic test. The increased sensitivity to detect organisms in the GI Panel may account for the increased utilization of definitive antimicrobials in the post-implementation cohort.

**Disclosures:**

J Kristie Johnson, PhD, D (ABMM), biomerieux: has given presentations/speeches for the company listed above Kimberly C. Claeys, PharmD, PhD, bioMérieux: Advisor/Consultant|bioMérieux: Honoraria

